# Assessing the Yield Potential and Ratooning Capacity of High‐Yielding Bangladeshi Rice Cultivars

**DOI:** 10.1155/sci5/6729630

**Published:** 2026-11-13

**Authors:** Md Masud Rana, Mirza Hasanuzzaman, P. V. Vara Prasad

**Affiliations:** ^1^ Department of Agronomy Bangladesh Agricultural University Mymensingh 2202 Bangladesh bau.edu.bd; ^2^ Department of Agronomy Faculty of Agriculture, Sher-e-Bangla Agricultural University Dhaka 1207 Bangladesh; ^3^ Department of Agronomy Kansas State University Manhattan 66506 Kansas USA k-state.edu

**Keywords:** genotypes, inbred, ratoon height, ratooning, yield components

## Abstract

In modern rice farming, identifying and promoting rice varieties with high yield in the main season and superior ratooning capacity for a second harvest can help enhance productivity, efficiency, and resilient cropping systems. This study evaluated the growth, yield, and adaptability of 34 inbred rice cultivars, including an advanced line, across main and ratoon seasons during the irrigated (*Boro*) season. Ratooning ability was tested at a 20 cm stubble height, showing significant differences among cultivars. Main crop yields ranged from 4.412 t ha^−1^ for BRRI dhan28 to 8.128 t ha^−1^ for BRRI dhan102, with BRRI dhan102 as the highest‐yielding cultivar, driven by superior tiller number (TN), panicle number (PN), leaf area index (LAI), and grain number (GN). Grain yield (GY) was positively correlated with PN, GN, LAI, and growth duration, but negatively with thousand‐grain weight. Multivariate analyses pointed out BRRI dhan102, BRRI dhan92, and BRRI dhan29 as high‐potential cultivars. Ratooning potential varied; 11 cultivars showed moderate to high potential, ranging from 0.78 to 1.50. BRRI dhan28, BRRI dhan81, BRRI dhan97, Bangabandhu dhan100, and Tata Miniket all reached ratoon yields over 1.00 t ha^−1^. The analysis of total yield across main and ratoon seasons showed highly significant differences among cultivars (*p*<0.001), with BRRI dhan102 (8.637 t ha^−1^) and Tata Miniket (8.495 t ha^−1^) producing the highest yields, indicating their superior productivity. The economic analysis reveals that cultivars such as BR17, BRRI dhan97, and Tata Miniket offer the highest net returns and benefit–cost ratios during both main and ratoon seasons, indicating their superior profitability and efficiency for rice cultivation. Further research through multilocation tests and under different management practices can identify the potential of these lines for use in the breeding program to develop sustainable rice production systems suitable for Bangladesh.

## 1. Introduction

Rice (*Oryza sativa* L.) is the staple food of Bangladesh and plays a pivotal role in national food security, rural livelihoods, and agricultural sustainability [1]. With increasing population pressure and shrinking opportunities for horizontal expansion of cultivated land, enhancing rice productivity through efficient land use and improved cropping systems has become a major research priority [2]. Bangladesh primarily follows three rice‐growing seasons: *Aus*, *Aman*, and *Boro*. Among the various cropping patterns, the *Boro*–Fallow–T. *Aman* system is one of the major rice‐based practices, accounting for approximately 26.92% of the country’s total cropped area [3]. In this system, the land generally remains fallow between the *Boro* harvest and the transplanting of T. *Aman*. This intervening period represents a potential opportunity to intensify rice production through ratoon rice cultivation without establishing a completely new crop. Developing a productive ratoon rice system within this fallow window may contribute to increased annual rice production, improved land‐use efficiency, and greater profitability without requiring the full range of inputs associated with conventional transplanted rice cultivation.

Traditional transplanted rice cultivation involves raising seedlings in a nursery, preparing the main field, transplanting seedlings, and managing the crop until maturity. Although this method is well established and supports high grain yields (GYs) under favorable conditions [4], it requires substantial labor, water, seed, and time for land preparation and crop establishment. In contrast, ratoon rice is produced from the regrowth of dormant or viable buds on the stubble remaining after harvesting the main crop. It eliminates the need for a second round of nursery establishment, seedling transplanting, and intensive land preparation, thereby offering a relatively rapid and resource‐efficient means of obtaining an additional rice harvest [5–7].

The process of producing a second rice (referred to as ratoon rice) from tillers that arise from nodal buds of the stubble of previously harvested main rice (main crop) is known as “ratoon rice” [6, 8]. Compared with planting a new crop, ratoon rice generally requires fewer resources and has a shorter growing duration while still producing an economically viable second harvest. Previous studies have shown that the success of ratoon rice production largely depends on the cultivar’s ratooning ability (RA), with substantial variation reported among rice genotypes [7]. Therefore, identifying cultivars with superior RA is a fundamental step toward developing efficient ratoon rice production systems.

The ratoon rice production system holds great promise for increasing productivity with lower input and labor requirements. In comparison to other multiple cropping systems, such as double rice season, ratoon rice can be grown in a shorter growing period with 50%–60% fewer inputs, little cost (such as labor, fertilizer, and water costs), and less land preparation, transplanting, and crop management, including pesticide application than the main crop [7], which offers an opportunity to increase cropping intensity with substantial net economic benefits and a smaller environmental footprint [9] when compared to other multiple cropping systems, such as double rice season.

In Bangladesh, economic development has exacerbated labor shortages in rural areas. Ratoon rice has emerged as a significant benefit for many marginalized farmers in Bangladesh, although it has not received much research attention. The typical *Boro*‐Fallow‐T. *Aman* cropping pattern practiced by farmers in Bangladesh involves transplanting *Aman* rice from the third week of July to the last week of August [10]. In this system, the field usually remains fallow for 2–3 months after harvesting *Boro* rice before being prepared for T. *Aman* rice, creating an opportunity to implement ratoon rice during the fallow period. Some farmers practice ratoon rice after harvesting *Boro* rice, which requires 50 to 60 days. The 60‐day ratoon growth duration of the rice cultivar can well fit into the subsequent cropping sequence.0

Although the yields of ratoon rice are relatively low (1.0–1.5 t ha^−1^) compared to the main crop, the associated costs are minimal [11]. By applying proper agronomic practices, such as deep urea placement during the main crop harvest and split applications of 1/4 urea and 1/3 MoP (muriate of potash) 10–12 days postharvest, ratoon crop yields can potentially increase to between 1 and 2 t ha^−1^ [11]. Ratoon technology can be extended to 10% of Bangladesh’s area where *Aus* cultivation or other crops are not viable due to early flooding. Cultivars that combine high main‐crop yield with rapid and vigorous tiller regeneration, early ratoon maturity, and satisfactory ratoon grain yield (RY) may offer considerable advantages for fitting an additional harvest into the fallow period of Bangladesh’s rice‐based cropping systems.

However, in Bangladesh, ratooning is underexplored, especially with new high‐yielding rice varieties that exhibit resilience to climate change and improved nutritional content. Identifying and promoting rice varieties with high yield in the main season and superior ratooning capacity can enhance productivity for the farming communities of this country.

The aims of this study were to (1) explore the agronomic output, growth, yield, and adaptability of thirty‐four inbred rice cultivars in the main season and (2) identify rice cultivars having RA and other desirable agronomic traits. The results of the present study could guide agricultural researchers in identifying the most promising candidates for further studies and breeding programs for developing sustainable rice production systems in the irrigated ecosystem of Bangladesh.

## 2. Materials and Methods

### 2.1. Description of Experimental Site

The experiment was conducted in the research field commonly known as “West Byde” of the Bangladesh Rice Research Institute (BRRI), Gazipur (90°33′E longitude and 23°77′N latitude), from November 2023 to September 2024 (Figure 1). The pH (6.82) of the experimental field soil was more or less neutral, with low organic matter content and fertility. The medium‐high land with a silty loam texture was characteristic of the research field land. The soil characteristics of the experimental field are shown in Table 1. The present study area was under a subtropical climate, where high temperatures and heavy rainfall were characteristic during April to September, and nominal rainfall was associated with moderately low temperatures from October to March (Figure 2).

**FIGURE 1:**
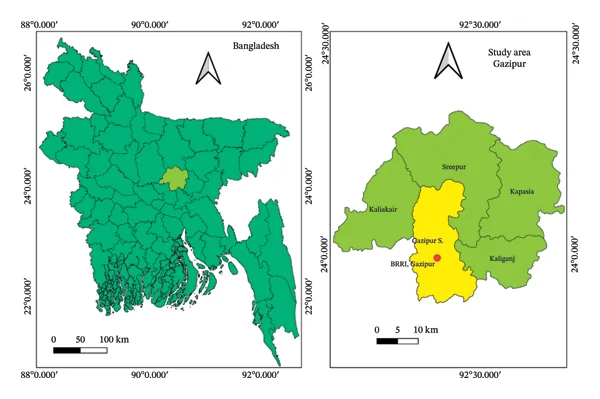
Study area map with reference to Bangladesh and the region.

**TABLE 1:** Soil nutrient status of the experimental site.

Nutrient	Value
pH	6.82
Organic matter (%)	2.13
Total *N* (%)	0.12
P (ppm)	18.21
K (meq 100 g^−1^ soil)	0.15
S (ppm)	9.81
Zn (ppm)	2.52

**FIGURE 2:**
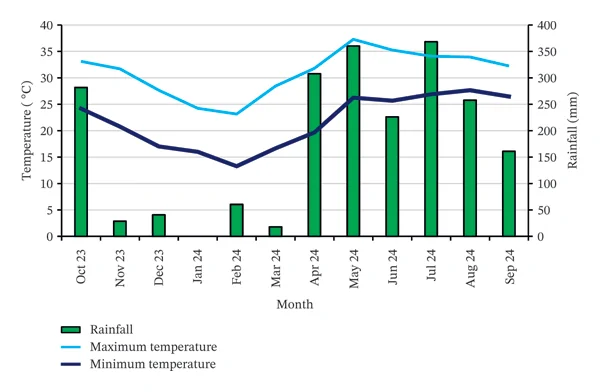
Temperature and rainfall variation during the experimental period in Gazipur, Bangladesh.

### 2.2. Planting Materials

Thirty‐four rice cultivars with different genetic backgrounds were used in this experiment (Table 2).

**TABLE 2:** List of parent materials with their key features.

Sl. No.	Variety	Growth duration (days)	Yield potential (t ha^−1^)	Special character
1	BR3	170	6.5	
2	BR16	165	6.0	Low GI
3	BR17	155	6.0	
4	BRRI dhan28	140	6.0	
5	BRRI dhan29	160	7.5	
6	BRRI dhan47	145	6.0	Salinity tolerant (6 dS m^−1^)
7	BRRI dhan50	155	6.0	Premium quality
8	BRRI dhan58	150	7.2	
9	BRRI dhan67	145	6.0	Salinity tolerant (8 dS m^−1^)
10	BRRI dhan74	147	7.1	Bio‐fortified rice (24.2 mg Zn/kg rice)
11	BRRI dhan75	115	5.5	Premium quality (*Aman* variety)
12	BRRI dhan81	143	6.5	Zira‐type grain, high protein
13	BRRI dhan84	141	6.5	Bio‐fortified rice (27.6 mg Zn kg^−1^ rice)
14	BRRI dhan86	145	6.5	Premium quality
15	BRRI dhan87	127	6.5	*Aman* season variety
16	BRRI dhan88	142	7.0	
17	BRRI dhan89	156	8.0	
18	BRRI dhan92	160	8.4	
19	BRRI dhan96	145	7.0	
20	BRRI dhan97	152	4.9	Salinity tolerant (8–10 dS m^−1^)
21	BRRI dhan98	112	5.8	*Aus* (heat tolerant)
22	BRRI dhan99	155	5.4	Salinity tolerant (8–10 dS m^−1^)
23	Bangabandhu dhan100	148	7.7	Bio‐fortified rice (25.7 mg Zn kg^−1^ rice)
24	BRRI dhan101	142	7.7	Disease tolerant
25	BRRI dhan102	150	8.10	Bio‐fortified rice (25.5 mg Zn kg^−1^ rice)
26	BRRI dhan104	147	7.3	Premium quality
27	BRRI dhan105	148	7.6	Low GI rice
28	Bina dhan‐5	150	6.0	
29	Bina dhan‐10	130	5.5	Salinity tolerant (10–12 dS m^−1^)
30	Bina dhan‐24	145	6.5	
31	Bina dhan‐25	138	7.6	Premium quality
32	Naria Choddo	155	6.8	Late *boro*
33	Tata Miniket	160	7.0	Premium quality
34	BR (Bio)15086‐AC16‐3‐21	165	8.0	

### 2.3. Trial Design and Main Crop Management Practices

The trial was laid out in a randomized complete block design with three replications. The layout of the experiment was done before transplanting, with interplot spacing of 50.0 cm and interblock spacing of 1.0 m. The size of each unit plot was 4.0 *m* × 2.5 m. Using 2–3 seedlings, 35‐day‐old seedlings were transplanted at 20 cm × 20 cm spacing. The BRRI‐recommended fertilizer doses for N, P, K, S, and Zn were applied at 120, 18, 75, 38, and 3.5 kg ha^−1^, respectively, through broadcasting during final land preparation, except urea. Urea was top‐dressed in three equal splits at 15, 30, and 45 days after transplanting. Weed and water management techniques were implemented as per the recommendations and crop requirements. The main crop was harvested at approximately 90% panicle maturity by uniformly cutting the plants at a stubble height of 20 cm above the soil surface.

### 2.4. Ratoon Crop Management Practices

Three days after harvest, irrigation (2–3 cm water depth) was applied and maintained for 1 week to promote ratoon regeneration. Weed control was initiated three days after harvesting (DAH) of the main crop with Granite GR (Penoxsulam) at 95 mL ha^−1^, followed by a single hand weeding at 30 DAH. Additionally, urea was applied at 30 kg N ha^−1^ immediately after the main crop harvest to stimulate tillering, followed by mulching (which involved stirring the soil surface with a weeding implement to incorporate the fertilizer into the soil). No phosphorus or potassium fertilizers, or other nutrients, were applied during the ratoon crop, as residual nutrients were sufficient due to the short duration. No additional irrigation was required, as the crop relied on rainfall during May–June. Insect control was achieved with a single application of Suntap Plus 50WP (containing 47.5% Cartap hydrochloride and 2.5% Fipronil), and brown spot disease was managed with Tilt 250EC.

### 2.5. Sampling Techniques, Yield Estimation During the Main Season

a. *Leaf area.* At 60 days after transplanting, four representative rice plants were randomly selected from each plot, and their leaves were carefully separated. The leaf area of each leaf was measured using a LiCOR 3100C Leaf Scanner, ensuring accurate and standardized assessments. The total leaf area per plant was then calculated by summing the individual leaf measurements.
b. *Plant height.* Plant height was measured at 90% panicle maturity by recording the length from the ground level to the tip of the panicle for five randomly selected hills per plot; the average height of these hills was used as the representative plant height for each plot.
c. *Growth duration.* Growth duration was determined by recording the number of days from seeding until the crop reached 90% maturity.
d. *Yield components.* Five destructive sample hills were randomly collected from each distinct plot outside the harvested region to measure key yield components. The total and effective tillers hill^−1^ were determined by counting all tillers from each of the five sampled hills and calculating their averages. The number of panicles was identified as those bearing flowers, while non‐effective tillers were counted separately from the remaining tillers and averaged across the samples. The number of grains panicle^−1^ was assessed by counting the grains on each panicle obtained from the same sampled hills, followed by averaging these counts to obtain representative values. Additionally, grains from the sampled panicles were sun‐dried in the open air to achieve a constant weight. The weight of 1000 grains was then recorded by randomly selecting and weighing a subsample of grains, providing the 1000‐grain weight measurement.
e. GY*:* Five square meters in the center of the plot were harvested at 90% panicle maturity without the use of border rows to quantify GY. The GY was adjusted to a moisture content of 14% and expressed in tons ha^−1^ after harvesting.

### 2.6. Sampling Techniques, Yield Estimation During the Ratoon Season

a. *Ratoon height.* Ratoon height was measured at 90% panicle maturity of ratoon crop by recording the length from the ground level to the tip of the panicle for five randomly selected hills plot^−1^; the average height of these hills was used as the representative plant height for each plot.
b. *Rarooning ability.* RA was measured as a ratio of total ratoon tillers to main crop tillers.
c. *Growth duration.* Ratoon growth duration was determined by recording the number of days from the main crop harvest until 90% maturity of the ratoon grain was achieved.
d. *Ratoon yield:* Five square meters in the center of the plot were harvested at 90% panicle maturity without the use of border rows to quantify RGY. The RGY was adjusted to a moisture content of 14% and expressed in tons ha^−1^ after harvesting.

### 2.7. Statistical Analysis

The analysis of variance (ANOVA) was performed in RStudio (Version 4.2.3) using the “doebioresearch” package. The mean values of cultivars were analyzed with the least significant difference (LSD) test at 5% probability levels. Heatmap, principal component analysis (PCA), Pearson’s correlation coefficient, and clustering were analyzed using the “ggplot2,” “metan,” “FactoMineR,” “factoextra,” and “cluster” packages. The study area map was created using “QGIS” software (Version 3.22.9).

## 3. Results

### 3.1. Yield of Rice Genotypes in Main Season

Different cultivars exhibit varying degrees of performance for various agronomic traits and GY. Among the 34 rice cultivars, BR17 and Bina dhan‐25 showed the tallest plant height, and BR3, BRRI dhan50, BRRI dhan81, BRRI dhan86, BRRI dhan88, and BRRI dhan96 showed the shortest plant height (Figure 3; Supporting Table 1). The highest number of tillers was demonstrated by BRRI dhan28, BRRI dhan67, BRRI dhan97, BRRI dhan102, and BRRI dhan104, and the lowest number of tillers by BR (bio) 15086‐AC16‐3‐21, BR17, Tata Miniket, BRRI dhan50, BRRI dhan75, and BRRI dhan101. The highest leaf area index (LAI) was observed in BRRI dhan102, followed by BRRI dhan50, BRRI dhan89, BRRI dhan97, BRRI dhan101, Naria choddo, and Tata Miniket. The lowest LAI was found in BR3, BR17, BRRI dhan81, BRRI dhan95, and Bina dhan‐24 (Supporting Table 1).

**FIGURE 3:**
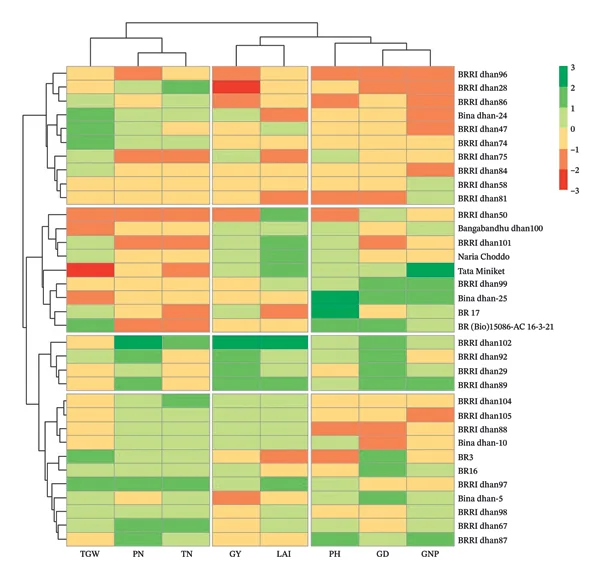
Comparison of yield and different yield‐contributing traits of thirty‐four rice cultivars.

The highest number of panicles was found in BRRI dhan102, followed by BRRI dhan67, BRRI dhan87, BRRI dhan89, BRRI dhan92, and BRRI dhan97, while the lowest number of panicles was observed in BRRI dhan50, BRRI dhan75, BRRI dhan96, BRRI dhan101, and BR (bio) 15086‐AC16‐3‐21. The superior number of grains panicle^−1^ was observed in Tata Miniket, and the inferior number of grains panicle^−1^ was observed in Bina dhan‐24, BRRI dhan28, BRRI dhan47, BRRI dhan84, BRRI dhan86, BRRI dhan96, and BRRI dhan105. The superior thousand‐grain weight was found in BR3, BRRI dhan47, BRRI dhan74, BRRI dhan97, BR (bio) 15086‐AC16‐3‐21, and Bina dhan‐24, while the inferior was observed in Tata Miniket, followed by Bina dhan‐25. BR3, BR16, BRRI dhan29, BRRI dhan89, BRRI dhan92, BRRI dhan99, BRRI dhan102, Bina dhan‐5, and Bina dhan‐25 were the long‐duration rice cultivars, whereas BRRI dhan28, BRRI dhan81, BRRI dhan88, BRRI dhan96, BRRI dhan101, and Bina dhan‐10 were the short‐duration cultivars (Supporting Table 2).

The *Scott–Knott* test results (*p* < 0.001) indicate significant differences among cultivars for main season yield. Cultivars such as BRRI dhan102 (8.128 t ha^−1^), BRRI dhan89 (7.079 t ha^−1^), and BRRI dhan92 (7.569 t ha^−1^) formed the high‐yielding group, showing statistically superior performance. Conversely, BRRI dhan28 (4.412 t ha^−1^) and Bina dhan‐5 (5.158 t ha^−1^) exhibited the lowest yields, classifying them as low‐yielding cultivars. Most cultivars clustered in the middle range with overlapping groups, suggesting variability within the moderate yield spectrum (Figure 3).

### 3.2. Correlation Among Yield and Yield Contributing Traits

The estimates of correlation coefficients computed between 12 characters of rice cultivars under the study area were presented in Figure 4. There was a negative and significant association between thousand‐grain weight and LAI (−0.32). Grain number (GN) had a significant and positive association with plant height (0.44), growth duration (GD) (0.60), and GY (0.27). On the other hand, GY had a significantly positive association with LAI (0.31), plant height (0.29), panicle number (PN) (0.23), and GD (0.28). GD and plant height (0.34), and PN and tiller number (TN) (0.51) were positively and significantly associated.

**FIGURE 4:**
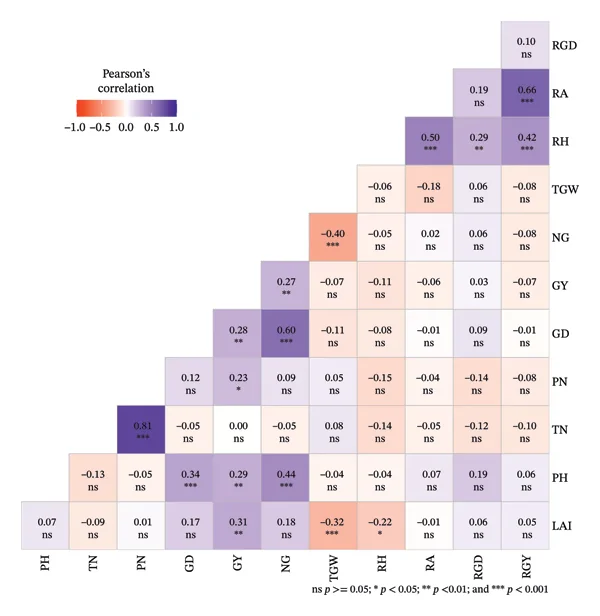
Correlation coefficient of yield and yield‐contributing traits. PH: plant height; TN: tiller number; PN: panicle number; GD: growth duration; GY: grain yield; NG: grain number; TGW: thousand grain weight; LAI: leaf area index; RH: ratoon height; RA: ratooning ability; RGD: ratoon growth duration; RGY: ratoon grain yield.

### 3.3. PCA

Table 3 shows the distribution of explained variance for each principal component (PC) by graphically representing the relationship between explained variance and the respective PC numbers. The first PC, denoted as PC1, accounted for 27.96% of the explained variance with an eigenvalue of 3.36, which subsequently declined gradually (Table 3). The graphical representation distinctly illustrates that PC1 accounts for the maximum variation among all PCs, with notable positive contributions observed for GY (0.677), GD (0.723), plant height (0.650), LAI (0.553), number of grains (0.696), ratoon height (0.766), and ratoon growth duration (0.539). Furthermore, the second PC, denoted as PC2, had an eigenvalue of 2.79 and explained 23.26% of the total variance associated with the identified yield component variables, specifically TN (0.878), PN (0.948), RA (−0.696), and ratoon yield (−0.597). Furthermore, the third PC, designated as PC3, exhibited an eigenvalue of 1.73 and accounted for more than 14.40% of the overall variance, demonstrating a positive contribution to the thousand‐grain weight (0.693) and a negative contribution to LAI (−0.565). The first, second, and third PCs encompass variables related to phenological traits, yield, yield‐affecting factors, and grain characteristics. Consequently, selecting lines from PC1 is expected to yield advantageous outcomes (Table 3). Consequently, solely the initial three PCs are deemed pertinent for subsequent analysis. The PCA biplot clearly grouped the 34 rice varieties into three main categories according to their agronomic performance (see Figures 5 and 6). The rice varieties located in the positive quadrant of PC1 had a high GY, a greater number of grains per panicle, a higher LAI, were taller and had a longer growth period; this shows that they are high‐yielding types for main‐season rice and have a higher RA, longer ratoon height with longer growth duration, and finally produce higher ratoon yield for ratoon rice after the main season. On the other hand, the cultivars mainly situated on the positive side of PC2 had very good tillering capacity with a higher number of both productive and total tillers, even though their GY was relatively moderate.

**TABLE 3:** Contributions of principal component (PC) of 12 traits for 34 rice cultivars evaluated.

Traits	Principal components		
	PC1	PC2	PC3
GY	0.677	0.339	0.035
GD	0.723	0.091	0.327
PN	0.138	0.948	0.038
TN	−0.217	0.878	−0.029
PH	0.650	0.152	−0.272
LAI	0.553	0.288	−0.565
GNP	0.696	0.347	−0.356
TGW	−0.488	0.311	0.693
RH	0.766	−0.264	0.092
RA	0.508	−0.696	0.342
RGD	0.539	0.304	0.393
RGY	0.458	−0.597	0.477
% variance	27.96	23.26	14.40
Cumulative % of variance	27.96	51.22	65.62
Eigenvalues	3.36	2.79	1.73

**FIGURE 5:**
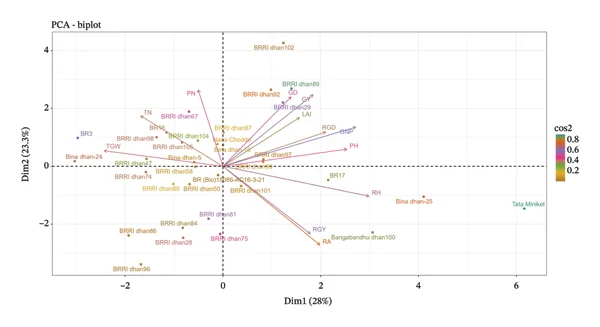
Principle component analysis (PCA) biplot based on principal component analysis of eight traits and thirty‐four rice cultivars. PH: plant height; TN: tiller number; PN: panicle number; GD: growth duration; GY: grain yield; GNP: grain number panicle^−1^; TGW: thousand grain weight; LAI: leaf area index; RH: ratoon height; RA: ratooning ability; RGD: ratoon growth duration; RY: ratoon yield.

**FIGURE 6:**
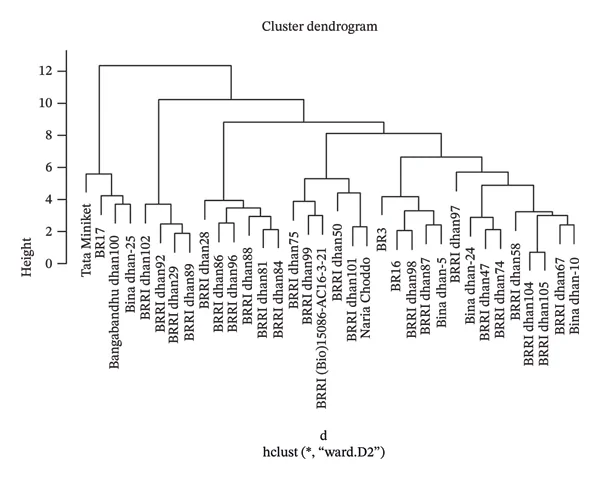
Clustering of thirty‐four rice cultivars established based on twelve traits.

Hierarchical cluster analysis supported the PCA classification by grouping the 34 cultivars into three distinct clusters. The hierarchical clustering analysis was instrumental in predicting and differentiating distinct groups predicated upon their characteristics within specific growing conditions. For clarity and prediction, the genetic traits were likewise categorized into three groups (Figures 5 and 6). To acquire a more in‐depth comprehension of the interrelations among these cultivars and their associated traits, they were subsequently depicted in a PCA biplot (Figure 5). The initial cultivars BRRI dhan102, BRRI dhan92, BRRI dhan29, and BRRI dhan89 were clustered in cluster 1 (Figure 6) and were established based on the traits PN, LAI, GNP, GD, and GY (Figure 5). These cultivars can therefore be regarded as elite, high‐yielding genotypes.

### 3.4. Performance of Ratoon Crops

The data presented in Tables 4 and 5 summarize the agronomic performance of various rice cultivars evaluated at BRRI, Gazipur, focusing on the ratoon crop. Key metrics include ratoon height, RA, GD, and yield. Ratoon height, which can affect the subsequent growth of ratoon crops, varied from 30.00 cm (BR3) to 73.00 cm (Bangabandhu dhan100). RA, measured as a ratio of total ratoon tillers to main crop tillers, indicates how well a cultivar can regenerate after the main harvest. There was considerable variation in RA between genotypes. Most cultivars showed relatively low RA, with several below 0.5. This suggests that while the main crop may yield well, the potential for subsequent ratoon crops varies significantly and may require careful selection of cultivars. Eleven screened cultivars (BR17, BRRI dhan28, BRRI dhan74, BRRI dhan81, BRRI dhan84, BRRI dhan86, BRRI dhan96, BRRI dhan97, Bangabandhu dhan100, Bina dhan‐25, and Tata Miniket) showed ratooning potential, with values of 0.78 to 1.50. Ratoon crop growth durations were generally shorter than those of main crops, resulting in low yield. Ratoon yields ranged from 0.236 t ha^−1^ to 1.553 t ha^−1^. Of the eleven genotypes, BRRI dhan28, BRRI dhan81, BRRI dhan97, Bangabandhu dhan100, and Tata Miniket were only capable of producing a ratoon yield of at least 1.00 t ha^−1^. Tata Miniket and BRRI dhan97 exhibit remarkable ratoon yield, indicating that they are particularly efficient at producing a second crop after the main harvest (Figure 7). High temperatures during harvesting of the main crop may accelerate reproductive growth and reduce the yield of the ratoon crop (Figure 8).

**TABLE 4:** Ratoon crop performance of the tested cultivars at BRRI, Gazipur.

Genotypes	Ratoon height (cm)	Ratooning ability	Growth duration (days)
BR3	30.00 e	0.48 c	39 c
BR16	48.00 c	0.50 c	34 d
BR17	64.33 a	0.78 c	51 b
BRRI dhan28	56.00 b	1.09 b	49 b
BRRI dhan29	49.33 c	0.36 c	60 a
BRRI dhan47	46.33 c	0.45 c	54 a
BRRI dhan50	37.00 e	0.46 c	42 c
BRRI dhan58	42.67 d	0.53 c	49 b
BRRI dhan67	47.00 c	0.33 c	49 b
BRRI dhan74	51.67 c	0.83 b	46 b
BRRI dhan75	58.67 b	0.76 b	35 d
BRRI dhan81	49.33 c	0.98 b	41 c
BRRI dhan84	49.33 c	0.96 b	35 d
BRRI dhan86	46.67 c	0.85 b	38 c
BRRI dhan87	46.33 c	0.56 c	34 d
BRRI dhan88	41.67 d	0.74 c	34 d
BRRI dhan89	45.67 c	0.42 c	57 a
BRRI dhan92	50.00 c	0.50 c	55 a
BRRI dhan96	37.67 e	1.02 b	41 c
BRRI dhan97	58.33 b	0.97 b	50 b
BRRI dhan98	49.00 c	0.40 c	31 d
BRRI dhan99	53.00 c	0.46 c	34 d
Bangabandhu dhan100	73.00 a	1.39 a	47 b
BRRI dhan101	45.00 c	0.61 c	41 c
BRRI dhan102	47.33 c	0.38 c	54 a
BRRI dhan104	44.00 c	0.72 c	46 b
BRRI dhan105	44.33 c	0.61 c	47 b
Bina dhan‐5	47.67 c	0.45 c	34 d
Bina dhan‐10	58.33 b	0.34 c	49 b
Bina dhan‐24	35.33 e	0.55 c	44 b
Bina dhan‐25	70.33 a	1.41 a	58 a
Naria Choddo	41.00 d	0.60 c	48 b
Tata Miniket	65.67 a	1.50 a	59 a
BR (Bio)15086‐AC16‐3‐21	51.67 c	0.40 c	33 d
Level of significance	^∗∗∗^	^∗∗∗^	^∗∗∗^
CV (%)	9.35	7.87	6.83

*Note:* Values with the same letter within the column are not statistically different (*Scott–Knott* test, *p* < 0.001). ^∗∗∗^indicates a 0.001 significance level of probability.

**TABLE 5:** Total yield of rice cultivars in main and ratoon seasons at BRRI, Gazipur.

Sl. No.	Cultivar	Yield (t ha^−1^)		
		Main season	Ratoon season	Total
1	BR3	5.626 b	0.236 c	5.861 b
2	BR16	6.357 a	0.397 c	6.754 b
3	BR17	6.950 a	0.965 b	7.915 a
4	BRRI dhan28	4.412 c	1.144 b	5.556 b
5	BRRI dhan29	7.404 a	0.410 c	7.814 a
6	BRRI dhan47	6.020 b	0.321 c	6.341 b
7	BRRI dhan50	4.905 c	0.556 c	5.460 b
8	BRRI dhan58	5.961 b	0.365 c	6.326 b
9	BRRI dhan67	6.136 b	0.418 c	6.554 b
10	BRRI dhan74	5.863 b	0.495 c	6.358 b
11	BRRI dhan75	6.336 a	0.709 c	7.044 a
12	BRRI dhan81	6.066 b	1.030 b	7.096 a
13	BRRI dhan84	5.610 b	0.937 b	6.547 b
14	BRRI dhan86	4.600 c	0.992 b	5.592 b
15	BRRI dhan87	6.001 b	0.561 c	6.562 b
16	BRRI dhan88	6.290 a	0.930 b	7.220 a
17	BRRI dhan89	7.079 a	0.499 c	7.578 a
18	BRRI dhan92	7.569 a	0.489 c	8.058 a
19	BRRI dhan96	5.221 c	0.947 b	6.168 b
20	BRRI dhan97	6.380 a	1.372 a	7.752 a
21	BRRI dhan98	5.782 b	0.261 c	6.043 b
22	BRRI dhan99	5.842 b	0.557 c	6.399 b
23	Bangabandhu dhan100	6.588 a	1.056 b	7.643 a
24	BRRI dhan101	6.470 a	0.553 c	7.023 a
25	BRRI dhan102	8.128 a	0.508 c	8.637a
26	BRRI dhan104	6.701 a	0.444 c	7.145 a
27	BRRI dhan105	6.490 a	0.480 c	6.970 a
28	Bina dhan‐5	5.158 c	0.755 c	5.912 b
29	Bina dhan‐10	6.716 a	0.428 c	7.145 a
30	Bina dhan‐24	6.199 b	0.406 c	6.605 b
31	Bina dhan‐25	5.929 b	0.668 c	6.597 b
32	Naria Choddo	6.851 a	0.498 c	7.349 a
33	Tata Miniket	6.943 a	1.553 a	8.495 a
34	BR (Bio)15086‐AC16‐3‐21	5.683 b	0.263 c	5.945 b
	Level of significance	^∗∗∗^	^∗∗∗^	^∗∗∗^
	CV (%)	11.08	7.36	10.62

*Note:* Values with the same letter within the column are not statistically different (*Scott–Knott* test, *p* < 0.001). ^∗∗∗^indicates a 0.001 significance level of probability.

**FIGURE 7:**
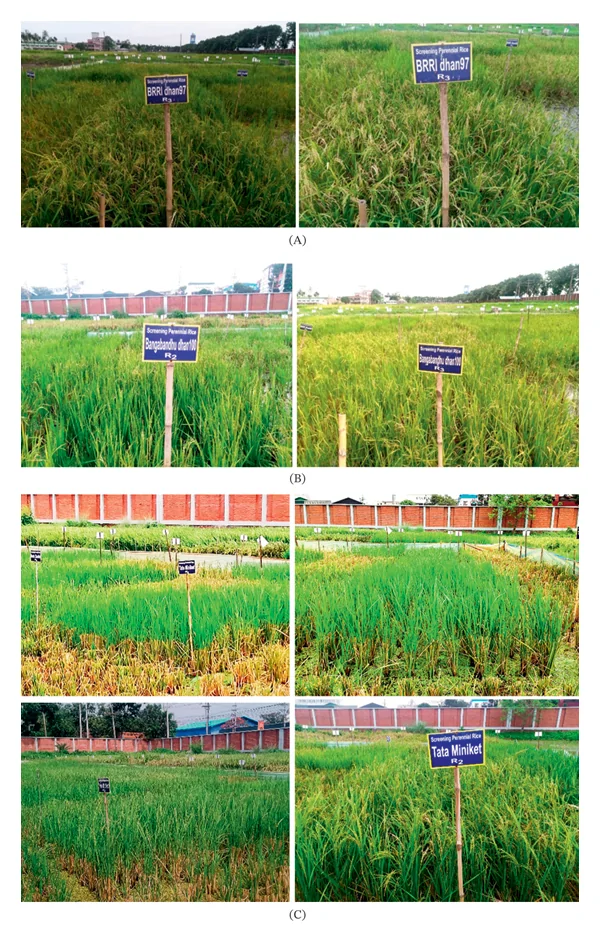
Ratoon crop performance of BRRI dhan97 (A), Bangabandhu dhan100 (B), and Tata Minket (C) at BRRI, Gazipur.

**FIGURE 8:**
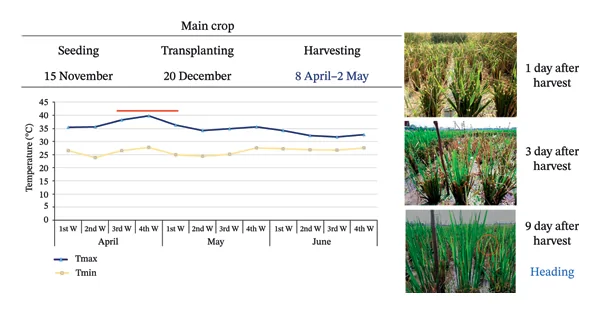
Effect of high temperature during harvesting of main crop on reproductive growth of ratoon crop.

The analysis of total yield across main and ratoon seasons revealed significant differences among the tested cultivars, as indicated by the highly significant level (*p* < 0.001). Based on *Scott–Knott* test, cultivars were grouped into different categories according to their mean yields. The highest yielding cultivar was BRRI dhan102, with a total yield of 8.637 t ha^−1^, significantly outperforming most other cultivars and forming the top group (Table 5). Close behind, Tata Miniket also demonstrated high productivity with 8.495 t ha^−1^, though it was statistically similar to BRRI dhan102. BRRI dhan89 (7.578 t ha^−1^), BRRI dhan97 (7.752 t ha^−1^), and Bangabandhu dhan100 (7.643 t ha^−1^) also showed good yields, but with overlapping groupings indicating no significant difference among them. Conversely, the lowest yielding cultivars were BR3 (5.861 t ha^−1^), BRRI dhan28 (5.556 t ha^−1^), Bina dhan‐5 (5.912 t ha^−1^), and BRRI dhan86 (5.592 t ha^−1^). These cultivars fell into the lower yield group, with some statistically similar to one another. Overall, the cultivars can be classified into three groups: a top group with BRRI dhan102 and Tata Miniket, a medium group including cultivars like BRRI dhan89 and BRRI dhan97, and a lower group comprising BR3, BRRI dhan28, and Bina dhan‐5. This grouping provides valuable insights for selecting high‐yielding cultivars for cultivation.

### 3.5. Economics of Rice Cultivars in Main and Ratoon Seasons

Table 6 presents an economic analysis of various rice cultivars across main and ratoon seasons, focusing on variable costs, gross returns, net returns, and benefit–cost ratios (BCR). Most of the cultivars incurred a consistent variable cost of Tk 97,800 ha^−1^, with additional seasonal costs of Tk 19,325 for ratoon crops. The majority of cultivars exhibit significantly higher gross and net returns during the main season compared to the ratoon season, reflecting the greater productivity and profitability of the initial crop. Cultivars such as Tata Miniket and BRRI dhan97 demonstrate notably high gross returns of Tk 49,696 and Tk 43,904 ha^−1^, respectively, resulting in strong net returns of Tk 30,371 and Tk 24,579 during the ratoon season. These cultivars also exhibit beneficial BCR of 2.57 and 2.27, indicating highly profitable ratoon crops. BR17, BRRI dhan28, BRRI dhan81, BRRI dhan84, BRRI dhan86, BRRI dhan88, BRRI dhan96, and Bangabandhu dhan100 also perform well, with BCR above 1.5, signifying good economic viability. Overall, cultivars such as Tata Miniket and BRRI dhan97 emerge as the most profitable options for ratoon cultivation.

**TABLE 6:** Economic performance of rice cultivars grown in the main and ratoon seasons.

Cultivar	Variable cost (Tk ha^−1^)		Gross return (Tk ha^−1^)		Net return (Tk ha^−1^)		Benefit–cost ratio	
	Main season	Ratoon season	Main season	Ratoon season	Main season	Ratoon season	Main season	Ratoon season
BR3	97,800	19,325	1,80,032	7552	82,232	−11773	1.84	0.39
BR16	97,800	19,325	2,03,424	12,704	1,05,624	−6621	2.08	0.66
BR17	97,800	19,325	2,22,400	30,880	1,24,600	11,555	2.27	1.60
BRRI dhan28	97,800	19,325	1,41,184	36,608	43,384	17,283	1.44	1.89
BRRI dhan29	97,800	19,325	2,36,928	13,120	1,39,128	−6205	2.42	0.68
BRRI dhan47	97,800	19,325	1,92,640	10,272	94,840	−9053	1.97	0.53
BRRI dhan50	97,800	19,325	1,56,960	17,792	59,160	−1533	1.60	0.92
BRRI dhan58	97,800	19,325	1,90,752	11,680	92,952	−7645	1.95	0.60
BRRI dhan67	97,800	19,325	1,96,352	13,376	98,552	−5949	2.01	0.69
BRRI dhan74	97,800	19,325	1,87,616	15,840	89,816	−3485	1.92	0.82
BRRI dhan75	97,800	19,325	2,02,752	22,688	1,04,952	3363	2.07	1.17
BRRI dhan81	97,800	19,325	1,94,112	32,960	96,312	13,635	1.98	1.71
BRRI dhan84	97,800	19,325	1,79,520	29,984	81,720	10,659	1.84	1.55
BRRI dhan86	97,800	19,325	1,47,200	31,744	49,400	12,419	1.51	1.64
BRRI dhan87	97,800	19,325	1,92,032	17,952	94,232	−1373	1.96	0.93
BRRI dhan88	97,800	19,325	2,01,280	29,760	1,03,480	10,435	2.06	1.54
BRRI dhan89	97,800	19,325	2,26,528	15,968	1,28,728	−3357	2.32	0.83
BRRI dhan92	97,800	19,325	2,42,208	15,648	1,44,408	−3677	2.48	0.81
BRRI dhan96	97,800	19,325	1,67,072	30,304	69,272	10,979	1.71	1.57
BRRI dhan97	97,800	19,325	2,04,160	43,904	1,06,360	24,579	2.09	2.27
BRRI dhan98	97,800	19,325	1,85,024	8352	87,224	−10973	1.89	0.43
BRRI dhan99	97,800	19,325	1,86,944	17,824	89,144	−1501	1.91	0.92
Bangabandhu dhan100	97,800	19,325	2,10,816	33,792	1,13,016	14,467	2.16	1.75
BRRI dhan101	97,800	19,325	2,07,040	17,696	1,09,240	−1629	2.12	0.92
BRRI dhan102	97,800	19,325	2,60,096	16,256	1,62,296	−3069	2.66	0.84
BRRI dhan104	97,800	19,325	2,14,432	14,208	1,16,632	−5117	2.19	0.74
BRRI dhan105	97,800	19,325	2,07,680	15,360	1,09,880	−3965	2.12	0.79
Bina dhan‐5	97,800	19,325	1,65,056	24,160	67,256	4835	1.69	1.25
Bina dhan‐10	97,800	19,325	2,14,912	13,696	1,17,112	−5629	2.20	0.71
Bina dhan‐24	97,800	19,325	1,98,368	12,992	1,00,568	−6333	2.03	0.67
Bina dhan‐25	97,800	19,325	1,89,728	21,376	91,928	2051	1.94	1.11
Naria Choddo	97,800	19,325	2,19,232	15,936	1,21,432	−3389	2.24	0.82
Tata Miniket	97,800	19,325	2,22,176	49,696	1,24,376	30,371	2.27	2.57
BR (Bio)15086‐AC16‐3‐21	97,800	19,325	1,81,856	8416	84,056	−10909	1.86	0.44

## 4. Discussion

### 4.1. Effects on Yield and Yield Traits of Rice Cultivars

The current study revealed significant genotypic variability among the 34 rice cultivars across key agronomic and yield‐contributing traits, highlighting the potential for targeted breeding and selection for high‐yielding ratoon rice. Such phenotypic diversity underpins the capacity of rice cultivars to respond differentially to agro‐ecological conditions and management practices, as also reported in earlier studies by References [12–14]. The tallest plants suggest potential advantages such as deep‐water or flood‐prone environments and biomass production [13, 15]. Conversely, cultivars exhibited shorter stature, which could be beneficial for lodging resistance and suitable for high‐input, high‐density planting systems [15]. The number of tillers plant^−1^ and panicles plant^−1^ are critical components of GY architecture. High tiller‐producing cultivars such as BRRI dhan28, BRRI dhan67, BRRI dhan97, BRRI dhan102, and BRRI dhan104 demonstrated superior capacity for reproductive architecture formation. Moreover, not all high‐tiller‐producing cultivars translated this trait into higher PN or GY, which suggests a possible trade‐off with tiller mortality or nonproductive tillers [13, 16]. Interestingly, BRRI dhan102 maintained the highest LAI, reflecting an efficient source‐sink balance and strong photosynthetic capacity, ultimately resulting in high GY (Figure 3). However, high LAI in BRRI dhan50 and BRRI dhan101 did not translate into high yields, which indicates that efficient grain‐filling processes do not accompany excessive leaf growth; it may lead to shading effects or assimilate competition. Conversely, low LAI in BR3 and BR17 is associated with poor yield performance, highlighting the crucial role that leaf area plays in photosynthetic activity, supported by Reference [17]. BRRI dhan102 emerged as the most promising cultivar, integrating high LAI, PN, and GN, ultimately resulting in the maximum GY. Under ideal circumstances, this cultivar can be used as a donor parent or benchmark in breeding efforts seeking high production potential and may be included in ratoon rice cultivation.

### 4.2. Interrelationships of Yield Traits and Selection Strategy

The correlation matrix offers insights into trait associations relevant to the indirect selection of cultivars. GY showed positive and significant correlations with plant height, PN, LAI, GN, and GD, indicating the importance of structural, physiological, and reproductive traits in yield formation [13, 14, 18]. Notably, the significant negative correlation between thousand‐grain weight and both GN and LAI supports the presence of source‐sink trade‐offs. However, these antagonistic associations should be carefully considered during simultaneous trait improvement. PCA, PCA biplot, and cluster analysis were further conducted for in‐depth understanding.

### 4.3. Utility of Principal Component and Cluster Analysis and Selection Strategy

The application of PCA in this study provided a multivariate framework for unraveling the complex interrelationships among the agronomic traits across 34 rice cultivars. The first three PCs cumulatively explain 73.64% of the total phenotypic variation, indicating that the majority of variability among genotypes is captured, making them valuable prospects for selecting diverse parental lines [13, 14]. The first three PCs typically capture the most significant variation among accessions, and the traits correlated with these components are more useful for differentiating accessions [13, 19]. However, the PCA biplot (Figure 5) and hierarchical clustering (Figure 6) jointly offered a comprehensive view of trait interrelations and cultivar differentiation. The clustering pattern also emphasizes the genetic diversity present among the cultivar accessions [13, 14, 19]. The presence of distinct clusters suggests underlying genetic divergence, which maximizes heterosis in hybrid as well as inbred breeding programs [13, 14, 20].

Furthermore, the trait‐based clustering supports the PCA biplot interpretation by characterizing each major cultivar with the specific trait combinations that define each PC. A noteworthy association was found between Cluster 1, which included cultivars like BRRI dhan102, BRRI dhan92, BRRI dhan29, and BRRI dhan89, and high‐yielding traits including PN, GN, GD, LAI, and ultimately GY. The findings support the strategic role of PCA not only as a descriptive tool but also as a prediction framework for cultivar classification, trait prioritization, breeding decision‐making, and selection of cultivars under diverse conditions.

### 4.4. Performance of Ratoon Rice

The performance of rice cultivars as ratoon crops revealed substantial variability across all evaluated parameters, including ratoon height, RA, GD, and GY. This genotypic variation is a critical determinant of the adaptability of cultivars for ratoon cropping systems, especially under subtropical rice‐growing regions like those of Bangladesh. Ratoon height is frequently linked to potential biomass accumulation and the vigor of regrowth. In our study, ratoon heights varied greatly, ranging from 30.00 cm (BR3) to 64.33 cm (BR17), highlighting that cultivars differed significantly in their capacity for residual tillering and shoot regeneration [21, 22]. Ratoon height was comparatively higher in cultivars such as Tata Miniket, BRRI dhan97, and BRRI dhan75, which may enhance source‐sink connections for ratoon yield formation, because these cultivars exhibited the 2^nd^ highest LAI (Figure 3).

Ratooning ability is the ratio of ratoon tillers to main crop tillers, one important characteristic that indicates a cultivar’s capacity for vegetative regeneration. Our findings showed a wide variation of RA, with values ranging from 0.33 to 1.50. Shiraki et al. [23] also found a RA of 1.00. They demonstrated that genetic factors influence the tillering ability of ratoon crops, the growth conditions of the main crop, and crop management practices that affect photosynthetic ability. Tata Miniket (1.50), Bangabandhu dhan100 (1.39), and Bina dhan‐25 (1.41) all have threshold RA > 1.0, which is considered highly favorable and highlights prolific ratoon tillering (Table 4). Strong basal node vitality and delayed senescence are characteristics of these cultivars that improve axillary bud activation following the main crop harvest. On the other hand, several high‐yielding cultivars from the main crop (such as BRRI dhan102, BRRI dhan29, and BRRI dhan89) displayed a decreased ratooning capacity (< 0.5), which may be due to limitations from poor regrowth from cut stubble or architectural constraints. Plant height, leaf number, LAI, PN, panicle length, spikelet sterility percentage, and RA are highly influenced by ratoon yield [22]. However, the average yield of most cultivars was less than 1.0 t ha^−1^ in the ratoon crop’s GY performance.

Among the 34 rice cultivars, only Tata Miniket, BRRI dhan97, Bangabandhu dhan100, BRRI dhan28, and BRRI dhan81 exceeded 1.0 t ha^−1^, exhibiting respectable ratoon production. Interestingly, these cultivars also demonstrated moderate to high RA, highlighting that RA and ratoon yield are positively correlated. Furthermore, ratoon crops’ shortened growth period (averaging 35–50 days) may reduce biomass accumulation and panicle development, which could limit yield potential even though it is beneficial for rapid returns. Moreover, high ambient temperatures during the main rice harvest, with maximum temperatures over 31°C–35°C (Figure 8), probably accelerated the ratoon tiller’s transition from the vegetative to the reproductive phase, leading to partial grain filling and yield reductions. Spikelet sterility in rice during anthesis, when exposed to a 31°C maximum temperature, has severe effects around 33.7°C maximum temperature, while exposure to 31°C from 12 days before anthesis to 12 days following anthesis, spanning meiosis and early growth expansion, is responsible for high spikelet sterility [8, 24].

### 4.5. Economic Performance of Ratoon Rice

Most rice cultivars have a variable cost of about Tk 97,800 per hectare, with seasonal ratoon costs of Tk 19,325. The main season yields higher returns, with Tata Miniket and BRRI dhan97 achieving gross returns of Tk 49,696 and Tk 43,904, and net returns of Tk 30,371 and Tk 24,579. BCRs above 1.5 indicate profitable ratoon cultivation. Cost analysis reveals that ratoon cropping involves lower input costs compared to main season planting, primarily due to reduced seed and planting expenses. While main crops typically generate higher gross and net returns due to optimal growing conditions, ratoon crops offer a cost‐effective extension with additional income potential when managed properly. Overall, the data emphasize the importance of cultivar choice in optimizing returns, with certain varieties offering significant advantages for farmers seeking sustainable and profitable ratoon rice cultivation.

This study provides the first systematic evaluation of the ratooning potential of 34 rice cultivars under Bangladesh conditions and identified 11 promising cultivars for ratoon rice production. However, the findings should be interpreted considering several limitations. The experiment was conducted during a single *Boro* season at one location (BRRI, Gazipur), which may limit the broader applicability of the results. Only one stubble cutting height (20 cm) and nitrogen fertilizer were evaluated, whereas the effects of different cutting heights and balanced nutrient management were not investigated. Although daily temperature data were recorded, no correlation or regression analyses were performed to quantify the relationship between temperature, ratoon bud sprouting, and yield. Future multiseason and multilocation studies incorporating comprehensive environmental analyses and optimized crop management practices are needed to validate and refine these findings.

## 5. Conclusions and Future Direction

In conclusion, the performance of different rice cultivars varied for different agronomic traits and GY. In the main season, the higher tillering cultivars such as BRRI dhan102 produced a comparatively higher GY, followed by BRRI dhan29, BRRI dhan89, and BRRI dhan92. In contrast, there was a considerable variation in RA among the studied rice cultivars due to wide variations in plant characters and climatic factors. Among the thirty‐four rice, eleven cultivars (BR17, BRRI dhan28, BRRI dhan74, BRRI dhan81, BRRI dhan84, BRRI dhan86, BRRI dhan96, BRRI dhan97, Bangabandhu dhan100, Bina dhan‐25, and Tata Miniket) showed ratooning potentiality. Despite its high main‐season productivity, the low RA of BRRI dhan102 reduced its suitability for ratoon rice production, while Tata Miniket demonstrated superior RA and achieved the second‐highest total yield (8.495 *t* ha^−1^), making it the most suitable cultivar for sustainable ratoon rice cultivation. Climatic variations, including high postharvest seasonal temperatures, and GD negatively impact ratoon productivity. Future adaptive and field research under varied seasonal conditions and different management practices needs to focus on GD in ratoons, harvest cuts (height), and fertilizer management. The findings of this study can guide agricultural researchers in selecting the most effective rice cultivars for ratooning and for future use in developing high‐tillering and high ratoon yield cultivars.

## Author Contributions

Md Masud Rana: contributed to investigation, methodology, data curation, formal analysis, writing–original draft; Mirza Hasanuzzaman: responsible for conceptualization, methodology design, funding acquisition, and supervision of the overall project; P. V. Vara Prasad: involved in data validation, literature review, and manuscript editing.

## Funding

This study was supported by the U.S. Agency for International Development (USAID) under CFDA# 98.001—through the Feed the Future Sustainable Intensification Innovation Lab (cooperative agreement no. AID‐OAA‐L‐14–00006) at Kansas State University Contribution Number 27‐063‐J from the Kansas Agricultural Experiment Station.

## Disclosure

All authors have reviewed the final manuscript and agreed to be accountable for all aspects of the work presented.

## Conflicts of Interest

The authors declare no conflicts of interest.

## Supporting Information

Additional supporting information can be found online in the Supporting Information section.

## Supporting information


**Supporting Information 1** Supporting Table 1: Agronomic traits of 34 rice cultivars during the main season. Supporting Table 2: Growth duration and yield of rice cultivars in main season.

## Data Availability

The data that support the findings of this study are available from the corresponding author upon reasonable request.
