# Supplementary material for: Assessing the Yield Potential and Ratooning Capacity of High‐Yielding Bangladeshi Rice Cultivars
Source: Scientifica (Cairo). 2026 Nov 13;2026:6729630. doi: 10.1155/sci5/6729630 (PMC13768854; doi:10.1155/sci5/6729630)
Supplement: Supplementary file 1 [file SCI5-2026-6729630-s001.docx]

Supplementary Table 1: Agronomic traits of 34 rice cultivars during the main season.

| **Cultivar** | **LAI** | **Plant height (cm)** | **No. of tiller m^-2^** | **No. of panicle m^-2^** | **No. of grains panicle^-1^** | **1000-grain weight (g)** |
| --- | --- | --- | --- | --- | --- | --- |
| BR3 | 2.02 | 82.83 | 366.67 | 297.92 | 147.67 | 23.72 |
| BR16 | 2.69 | 89.42 | 366.67 | 310.42 | 193.10 | 22.16 |
| BR17 | 2.37 | 136.42 | 295.83 | 289.58 | 178.87 | 23.31 |
| BRRI dhan28 | 2.80 | 93.83 | 404.17 | 297.92 | 98.93 | 20.57 |
| BRRI dhan29 | 3.65 | 94.33 | 291.67 | 325.00 | 207.43 | 19.40 |
| BRRI dhan47 | 3.38 | 93.92 | 320.83 | 291.67 | 91.10 | 24.53 |
| BRRI dhan50 | 4.36 | 87.42 | 293.75 | 258.33 | 127.97 | 17.18 |
| BRRI dhan58 | 2.76 | 98.75 | 329.17 | 266.67 | 173.80 | 20.39 |
| BRRI dhan67 | 3.47 | 110.33 | 414.58 | 327.08 | 188.40 | 21.44 |
| BRRI dhan74 | 3.30 | 89.67 | 356.25 | 314.58 | 106.00 | 25.18 |
| BRRI dhan75 | 2.13 | 104.33 | 266.67 | 241.67 | 121.93 | 22.20 |
| BRRI dhan81 | 2.51 | 88.42 | 333.33 | 283.33 | 169.20 | 20.92 |
| BRRI dhan84 | 2.88 | 102.67 | 320.83 | 279.17 | 88.80 | 21.80 |
| BRRI dhan86 | 2.99 | 81.75 | 347.92 | 285.42 | 75.97 | 21.13 |
| BRRI dhan87 | 2.95 | 118.75 | 377.08 | 322.92 | 224.20 | 19.96 |
| BRRI dhan88 | 3.75 | 88.92 | 358.33 | 314.58 | 130.53 | 18.98 |
| BRRI dhan89 | 4.27 | 108.08 | 335.42 | 325.00 | 220.37 | 19.59 |
| BRRI dhan92 | 4.04 | 112.50 | 325.00 | 333.33 | 150.83 | 22.61 |
| BRRI dhan96 | 2.86 | 82.25 | 308.33 | 239.58 | 76.20 | 20.37 |
| BRRI dhan97 | 4.27 | 115.33 | 389.58 | 339.58 | 122.40 | 24.93 |
| BRRI dhan98 | 3.79 | 92.75 | 360.42 | 314.58 | 190.77 | 21.66 |
| BRRI dhan99 | 3.59 | 106.83 | 302.08 | 264.58 | 239.63 | 19.92 |
| Bangabandhu dhan100 | 3.81 | 108.58 | 308.33 | 264.58 | 165.27 | 17.85 |
| BRRI dhan101 | 4.59 | 116.58 | 275.00 | 245.83 | 137.43 | 21.54 |
| BRRI dhan102 | 5.46 | 104.75 | 402.08 | 354.17 | 193.20 | 19.89 |
| BRRI dhan104 | 3.92 | 102.83 | 383.33 | 297.92 | 108.33 | 20.24 |
| BRRI dhan105 | 3.61 | 100.67 | 379.17 | 308.33 | 100.83 | 20.19 |
| Bina dhan-5 | 3.17 | 115.08 | 375.00 | 277.08 | 184.03 | 22.40 |
| Bina dhan-10 | 3.38 | 104.75 | 358.33 | 304.17 | 150.10 | 20.12 |
| Bina dhan-24 | 1.92 | 93.92 | 356.25 | 318.75 | 83.17 | 25.23 |
| Bina dhan-25 | 2.78 | 132.00 | 302.08 | 270.83 | 236.37 | 17.64 |
| Naria Choddo | 4.23 | 111.33 | 327.08 | 268.75 | 140.33 | 23.36 |
| Tata Miniket | 4.46 | 113.58 | 258.33 | 270.83 | 296.20 | 13.62 |
| BR (Bio)15086-AC16-3-21 | 2.69 | 125.92 | 270.83 | 241.67 | 189.87 | 24.29 |
| Lsd (0.05) | 1.10 | 7.59 | 107.02 | 84.26 | 54.32 | 2.84 |

Supplementary Table 2: Growth duration and yield of rice cultivars in main season.

| **Sl. No.** | **Cultivar** | **Growth duration (days)** | **Yield**  **(t ha^−1^)** |
| --- | --- | --- | --- |
| 1 | BR3 | 168 | 5.626 |
| 2 | BR16 | 166 | 6.357 |
| 3 | BR17 | 158 | 6.950 |
| 4 | BRRI dhan28 | 148 | 4.412 |
| 5 | BRRI dhan29 | 167 | 7.404 |
| 6 | BRRI dhan47 | 153 | 6.020 |
| 7 | BRRI dhan50 | 162 | 4.905 |
| 8 | BRRI dhan58 | 157 | 5.961 |
| 9 | BRRI dhan67 | 155 | 6.136 |
| 10 | BRRI dhan74 | 153 | 5.863 |
| 11 | BRRI dhan75 | 154 | 6.336 |
| 12 | BRRI dhan81 | 151 | 6.066 |
| 13 | BRRI dhan84 | 153 | 5.610 |
| 14 | BRRI dhan86 | 152 | 4.600 |
| 15 | BRRI dhan87 | 163 | 6.001 |
| 16 | BRRI dhan88 | 149 | 6.290 |
| 17 | BRRI dhan89 | 166 | 7.079 |
| 18 | BRRI dhan92 | 169 | 7.569 |
| 19 | BRRI dhan96 | 145 | 5.221 |
| 20 | BRRI dhan97 | 158 | 6.380 |
| 21 | BRRI dhan98 | 158 | 5.782 |
| 22 | BRRI dhan99 | 166 | 5.842 |
| 23 | Bangabandhu dhan100 | 153 | 6.588 |
| 24 | BRRI dhan101 | 151 | 6.470 |
| 25 | BRRI dhan102 | 166 | 8.128 |
| 26 | BRRI dhan104 | 158 | 6.701 |
| 27 | BRRI dhan105 | 157 | 6.490 |
| 28 | Bina dhan-5 | 166 | 5.158 |
| 29 | Bina dhan-10 | 150 | 6.716 |
| 30 | Bina dhan-24 | 152 | 6.199 |
| 31 | Bina dhan-25 | 166 | 5.929 |
| 32 | Naria Choddo | 158 | 6.851 |
| 33 | Tata Miniket | 164 | 6.943 |
| 34 | BR (Bio)15086-AC16-3-21 | 165 | 5.683 |
|  | Lsd (0.05) | 6.83 | 1.117 |
